# Supplementary material for: When most fMRI connectivity cannot be detected: Insights from time course reliability
Source: PLoS One. 2024 Dec 13;19(12):e0299753. doi: 10.1371/journal.pone.0299753 (PMC11642907; doi:10.1371/journal.pone.0299753)
Supplement: S1 File — (DOCX) [file pone.0299753.s001.docx]

**Supporting Information**

**When most fMRI connectivity cannot be detected: insights from time course reliability**

**S1 Text. Methods**

**Participant and task selection**

This investigation originally involved three tasks: a verbal working memory task, a spatial working memory task, and a number Stroop task. The time courses for these tasks consisted of 1166 timepoints in total. To estimate head motion, we measured the largest possible Euclidian distance in the observed head motion time courses, utilizing all available 1166 timepoints. This method is considered conservative, as only 488 volumes were relevant for this analysis. For the analysis, we specifically selected the verbal working memory task because 50 out of the initial 67 individuals were able to deliver at least 93 correct responses out of 96, resulting in an average correct response rate of 99%. To ensure data accuracy, we included only those individuals with head motions less than 3 mm. It is important to note that this criterion of < 3mm is smaller than the voxel size (4 mm) being studied.

**Task**

We selected this task because filled delay tasks tend to demonstrate favorable to excellent test-retest reliability, regardless of the distractor task employed [1]. Furthermore, one might argue, that the large differences in response times that are due to the mixed character of the filled delay task may induce sufficient variability for within-subject reliability analysis. The conventional group test-retest reliability of the memory items reported excellent results (r = 0.76) [2]. However conventional test-retest reliability may be less relevant in this study.

**fMRI details**

MRI scans were performed on a 3 T Siemens Magnetom Skyra (Siemens Medical Systems, Erlangen, Germany) equipped with a 32-channel head coil. Structural images were obtained by means of a 3D-MPRAGE sequence (176 slices per slab, FOV = 256 mm, TR = 2530 ms, TE = 2.07 ms, TI = 900 ms, Flip angle = 9°, voxel size = 1 mm isotropic). Functional imaging data were obtained using a Siemens Grappa parallel acquisition scheme with pat factor 2; using following parameters Flip Angle 72 degrees, TR = 1240 ms, TE = 30 ms. Volume dimensions were 64*64*23, with voxel resolution 4*4*4 mm with a gap of 10%. In total 488 volumes were obtained per task per session.

The grey and white matter were segmented using FreeSurfer routines (FreeSurfer). Subsequently the Grey matter time courses were brought into FS average space using spherical alignment methods as available in FreeSurfer. Next 34 MNI coordinates that are believed to be essential for working memory were taken from a meta-analysis and brought into 2D FS average space using standard FreeSurfer command. We created a circle of 8 mm in diameter around the coordinate of interest on the 2D representation of the brain and extracted the time courses of interest which were subsequently averaged.

White matter, ventricle and head motion time courses underwent principal component analysis as available in the FreeSurfer package. All noise and grey matter time courses of interest were read into MATLAB for further analysis steps. The grey matter time courses were detrended, denoised and corrected for head motion within a GLM framework. The slow signal trend present in grey matter time courses was detected using the spm_filter.m routine of the SPM package (cycle = 128 seconds) that was incorporated into our custom pipeline (The Wellcome Centre for Human Neuroimaging). The noise regressors consisted of 5 principle “white matter” components and 5 principal “ventricle” components. The motion regressors included the first 2 principal components of the head motion data.

Co-registration, segmentation, and motion correction were performed prior to registration for slice time correction and motion correction of functional data. The head motion was estimated as the largest possible Euclidean distance in mm between points in space. The FreeSurfer (FS) pipeline consists of two sub-pipelines: one in 3D volume space and one in 2D mesh surface space. In this study, the 3D volume pipeline was used to co-register the functional images obtained in the test and retest runs with the structural image obtained in the test run. Additionally, the nuisance time courses of the white matter, ventricles, and head motion were extracted using the 3D pipeline. The 2D pipeline was used to align the grey matter time courses to the FS average space with high accuracy.

**Alignment**

Spherical alignment, as provided by the FreeSurfer package, was used in this study to align the functional data of the individuals and also to project the MNI cordites from a meta-analysis into fs average space [3]. This approach is still one of the most accurate registration methods available. An advantage of spherical alignment is that it creates high anatomical correspondence between individuals without the need to perform spatial smoothing of the functional data to force overlap. Spatial smoothing is a weighted averaging procedure that can average irrelevant time courses belonging to the white matter and dura mater with the grey matter time course when performed in 3D. Moreover, spatial smoothing might connect functional distinct areas located on opposing sides of a sulcus and potentially create functional connectivities that are not present in the true world in 3D data. This can corrupt the true connectivity estimates between brain nodes. By contrast spatial smoothing on the surface does not suffer from the previously described problems when applied conservatively. However, we have opted against spatial smoothing on the surface for reasons discussed in the section “Creation of Patches of Interest and Extraction of Grey Matter Time Courses”. In summary, the functional data from the test and retest runs were aligned with the 3D structural anatomy of the test run using commands from FS Fast. The quality of the alignment procedure was visually inspected in all cases, and no mismatches were observed. Next, the structural scans from the test run were automatically segmented into white and grey matter mass using the standard "recon all" pipeline of the FreeSurfer package. The resulting white and grey matter segmentations were visually inspected, and hand corrections were made when necessary. The corrected volumes were re-entered into the FreeSurfer pipeline to obtain accurate spherical representations of the cortex in native space. The corrected spheres were aligned with the FreeSurfer FS average brain using spherical alignment approaches. The resulting sphere reg files were used later to align the functional data from the test and retest runs to the common FS average mesh. The 3D white matter volumes were eroded with two iterations to avoid spatial overlap between grey matter and white matter time courses. Finally, the volumes of the ventricles were defined and used to obtain nuisance time courses.

**Creation of Patches of Interest and Extraction of Grey Matter Time Courses**

As mentioned, we did not use spatial smoothing as a preprocessing step. A recent study that investigated the use of spatial smoothing as a preprocessing step in ROI based functional connectivity analysis concluded “ Spatial smoothing has complex effects on the structure and properties of the networks, including possible over-emphasis of strong, short-range links, changes in the identities of hubs of the network, and decreased inter-subject variation. The ROI approach already includes averaging, independent of spatial smoothing. Therefore, there is no specific reason for applying spatial smoothing” [4]. Working memory experiments were used in this study to investigate the functional connectivity of frontal parietal systems. Instead of creating patches of interest based on activation maxima potentially present in our own sample, which could cause circularity, we selected 34 MNI coordinates of interest from a recently published meta-analysis focusing on the executive aspects of working memory. These coordinates were brought into FS average mesh space using the procedure described on the FreeSurfer homepage. In short, the 3D coordinates of the ALE meta-analysis were brought into 3D FS average space using the fslregister command. Then, the co-registered coordinates were projected onto the FS average mesh using the FreeSurfer mrivol2surf command. The relevant mesh elements were brought into MATLAB® format for further preprocessing using the FreeSurfer/MATLAB® command mri read. A circle with a diameter of 8mm was drawn around the relevant mesh element using the MATLAB® surfstat command SurfStatROI. The resulting vertices were aggregated into a patch of interest (POI), which was used to extract the time courses of the individuals under study. All the mesh time courses of a specific POI were averaged and used to estimate the connectivity among nodes, as well as the test-retest reliability. We used a diameter of 8mm which is roughly twice the size of our voxels. This approach mimics to a certain extent the determination of the ideal FWHM of a smoothing kernel on the 2D surface that is believed to be around 8mm [5,6].

**Within subject time course reliability**

It is well established that connectivity is estimated using Pearson correlations while attempts to estimate connectivity by means of ICC are not known to the authors. The estimation of the connectivity upper bound implies that time-course reliability is estimated with Pearson ρ correlations. In addition, one might argue that since the magnitude of time courses is arbitrary in nature it makes less sense to estimate time course reliability by means of intraclass correlations that depending on the version of the model react very sensitive to the magnitude of a score. It is common practice in multi session fMRI to subject time courses to z-transformations to compensate for the obvious differences in signal magnitude. However, estimating Pearson correlations from z- transformed data results in point estimates that are very close to ICC correlations since z transformations effectively pool the data that are used to estimate the mean and variance. For reasons of method consistency all reliability estimates including behavioral analysis were obtained by means of interclass correlations even if intraclass correlations are available as an alternative.

**Residual autocorrelations**

Residual autocorrelations may occur within a region when time courses are auto correlated to obtain test-retest reliability information and between regions when time courses are correlated to obtain connectivity information. Residual autocorrelations were removed from the time courses that were subjected to connectivity and reliability analysis assuming an AR(1) model.

Xt = φXt−1 + εt (1)

The model is estimated from the residual autocorrelations of the timeseries. Within this context, the error term at timepoint Xt is predicted from the previous error term Xt−1. The strength of the relation between Xt and Xt−1 is denoted by φ. Finally, εt symbolizes the uncorrelated white noise at timepoint t. Once φ is estimated from the residual time courses it can be applied to the observed time courses to obtain time courses that are not plagued by lag one auto correlations a procedure also known as pre whitening. In a next step the AR(1) corrected time courses were entered into the final reliability and connectivity analysis. In short, the weight of residual autocorrelation was estimated for every within subject time course combination and then applied to the time course combination of interest.


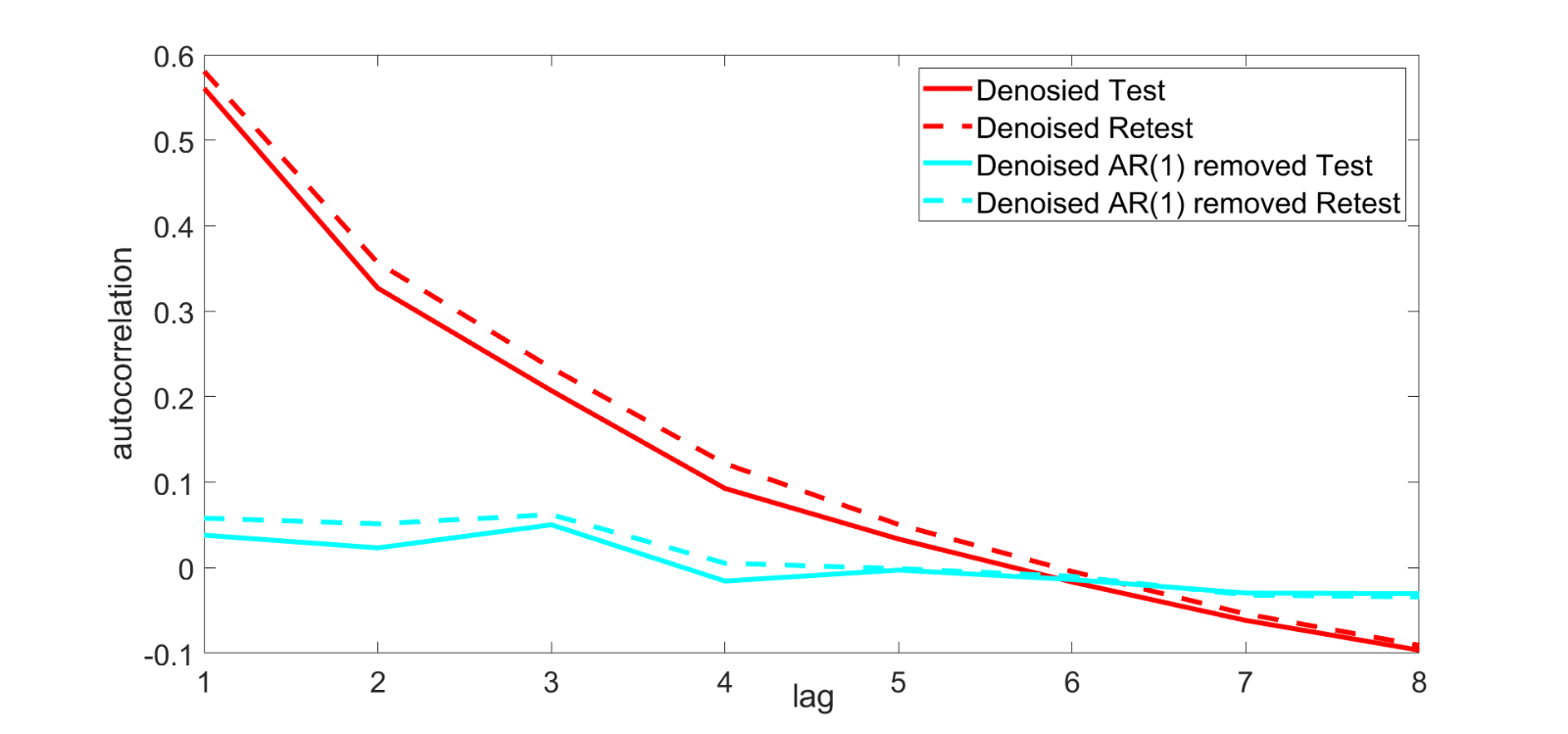


**In this analysis, the autocorrelation functions from denoised time courses were compared before and after eliminating serial autocorrelations. Autocorrelation functions of each individual time course were estimated for test and retest sessions and then averaged across the 34 regions* 50 individuals per session. Next, time courses of the test session were regressed with those of the retest session, and the beta weight from the lag 1 residual was applied to each respective time course. The resulting auto correlations functions per session were then averaged and plotted. Results suggest that node wise AR(1) corrections are effective albeit not perfect.**

We evaluated the effectiveness of the pre-whitening procedure by estimating the lag 1 to lag 8 autocorrelations of the denoised test-retest time courses before and after pre-whitening. This pre-whitening was performed as part of our test-retest reliability analysis. The results indicate that the standard pre-whitening approach successfully removed the initially high autocorrelations, though small autocorrelations remained at lags 1 to 3 not exceeding r = 0.05. Overall, this suggests pre-whitening is an effective method for reducing the high autocorrelation in the time courses, as intended, to facilitate correct test-retest reliability estimates while accounting for temporal dependencies in the signal. Similarly, to AFNI we estimate the weights that are used to correct for the autocorrelations at the level of the two time courses of interest which may be more accurate as other approaches although just like AFNI not perfect [7].

**S2 Text. Negative time course reliability**

The lower confidence interval of a zero test-retest reliability correlation may reach into the realm of negative correlations. Already in the mid-1950s, Cronbach argued that negative reliability is problematic [8]. Negative correlations do not reflect negative reliability - which implies some kind of coherent structure - but rather the complete absence of reliability. We treated negative test-retest reliability in several ways. For the naïve approach we simply averaged negative and positive correlations as well as negative and positive CIs. For the informed approach we only averaged positive correlations and CIs and in addition report the percentage of nodes that exhibited negative test-retest reliability and refer to this as the percentage of nodes with corrupt test-retest reliability. Finally, we also report sample statistics when negative correlations were set at zero. We conducted an analysis to understand the reasons behind the occurrence of negative test-retest reliability. Analysis provided in this section suggests that negative reliability is partly caused by a desynchronization of the test/retest time courses. We looked at the cross-correlation between the test run and the retest run. By determining the lag at which the highest test-retest reliability was achieved, we were able to identify the underlying factors. We compared the highest possible test-retest reliabilities obtained at different lags (depicted in yellow) with those observed at lag zero (depicted in blue). This was done for denoised data and data where the residual autocorrelations were removed. Our analysis, clearly showed that a significant portion of the negative test-retest reliability disappeared when non-zero lag cross-correlations were used to estimate reliability.


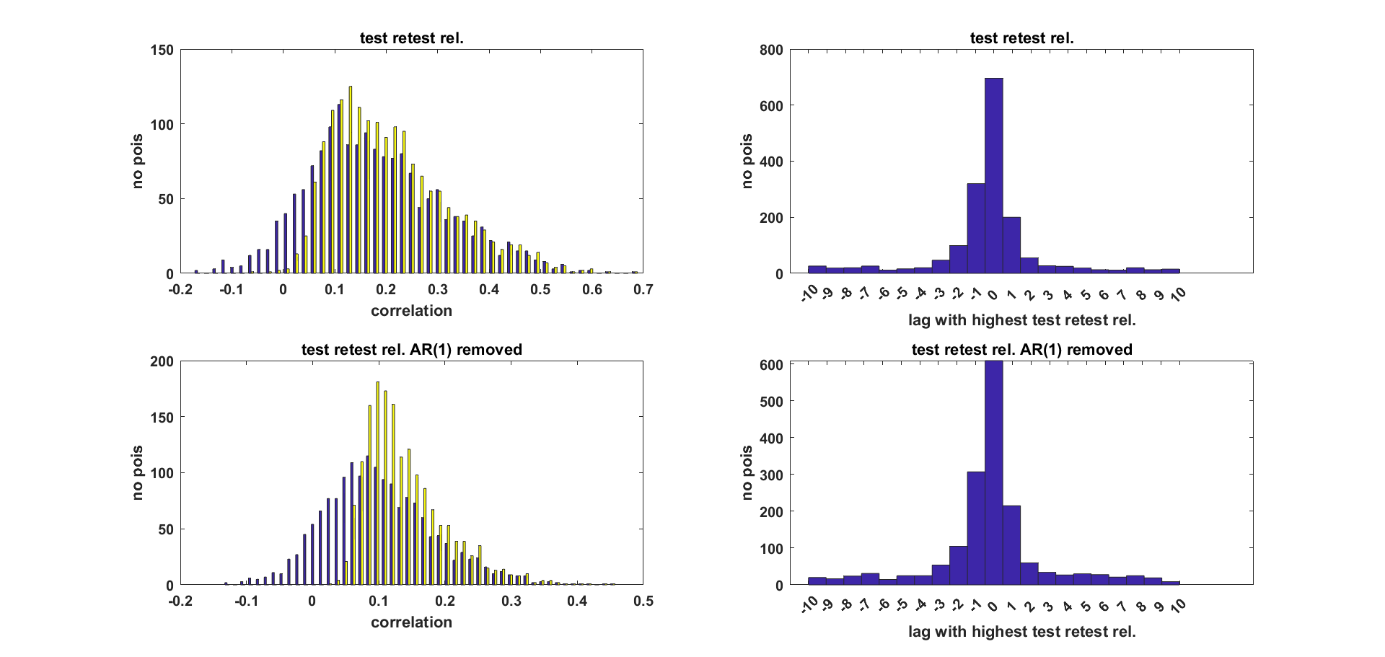
**The histograms depicted at the left visualize the distributions of the highest possible test-retest reliability obtained at different cross correlation lags in yellow while conventional test-retest reliability obtained at cross correlation lag zero was visualized in blue. The histograms depicted at the right reveal at which lag the highest test-retest reliability was observed. Mark that no pois refer to the number of patches of interest.**

Further investigation revealed that the majority of regions exhibited the highest test-retest reliability at lag zero. However, a substantial fraction of the time courses showed the highest reliability when shifted by one lag (lag -1 or lag 1). This was particularly noticeable for time courses with negative or close-to-zero reliability. Interestingly, we found that it was not possible to maximize test-retest reliability substantially in patches that already exhibited relatively high reliability, suggesting that desynchronization primarily affected time courses with poor reliability. Although it is feasible to estimate test-retest reliability of time courses for various lags this is not in line with the basic assumption that time courses should exhibit exactly the same behavior at the test and retest occasion. Hence, we preferred to set negative correlations to zero for some analysis. In neuro imaging two layers of statistics exist. Depending on the perspective - first or second level - one might treat negative test-retest reliability either as missing data or as a zero correlation. From a first level perspective one might argue that nodes or paths that are characterized by negative test-retest reliability do not exist in the single subjects under study which justifies to remove these nodes or paths from the analysis when estimating descriptive statistics. From a second-level perspective, it may be more appropriate to set nodes or paths with negative test-retest reliability to zero. This ensures that degrees of freedom remain constant across paths and measurement occasions when performing t-tests. For the first level perspective we computed the percentage of nodes that exhibited negative test-retest reliability and refer to this as corrupt reliability. Within this context, we estimated the mean reliability and the mean confidence intervals when correlations exhibiting negative test-retest reliability were omitted from the data. For the second level we replaced negative test-retest reliability by zero and averaged time course reproducibility estimates. It should be noticed that it is not possible to obtain confidence intervals for time course reliability estimates that were set at zero trough bootstrapping which is the reason why we do not give CI in this particular case. Finally, we estimated the confidence interval of the sample mean and CI of the sample standard deviation from the 34 nodes*50 subjects = 1700 reliability correlations estimated in the previous steps through bootstrapping. We estimated sample statistics for the data as observed and for the data where negative reliability was replaced by zero.

**S1 Table. Grand mean reliability**

| **OriginalVariableNames** | **reliability** | **reliability AR(1)** | **description** |
| --- | --- | --- | --- |
| Mean | 0.19 | 0.10 | grand mean within subject time course reliability negative values included |
| TimecourseLow | 0.07 | 0.01 | lower bound of within subject time course reliability negative values included |
| TimecourseUp | 0.29 | 0.19 | upper bound of within subject time course reliability negative values included |
| Distribution Percentile 2.5% | -0.04 | -0.04 | lower bound of the entire distribution negative values included |
| Distribution Percentile 97.5% | 0.47 | 0.29 | upper bound of the entire distribution negative values included |
| DistributionStd | 0.14 | 0.09 | Std of the entire distribution negative values included |
| DistributionMeanLow | 0.18 | 0.10 | lower bound of the mean negative values included |
| DistributionMeanUp | 0.19 | 0.11 | upper bound of the mean negative values included |
| ZeroMean | 0.19 | 0.11 | grand mean within subject time course reliability negative values replaced by zero |
| ZeroDistribution Percentile 2,5% | 0.00 | 0.00 | lower bound of the entire distribution negative values set at zero |
| ZeroDistribution Percentile 97.5% | 0.47 | 0.29 | upper bound of the entire distribution negative values set at zero |
| ZeroDistributionMeanLow | 0.14 | 0.08 | lower bound of the mean negative values set at zero |
| ZeroDistributionMeanUp | 0.18 | 0.10 | upper bound of the mean negative values set at zero |
| ZeroDistributionStd | 0.19 | 0.11 | Std of the entire distribution negative values set at zero |
| Corrupt | 6.24 | 9.41 | percentage of corrupt paths |
| NoZeroMean | 0.20 | 0.12 | grand mean within subject time course reliability negative values excluded |
| NoZeroTimecourseLow | 0.09 | 0.03 | lower bound of within subject time course reliability negative values excluded |
| NoZeroTimecourseUp | 0.31 | 0.20 | upper bound of within subject time course reliability negative values excluded |
| NoZeroDistribution Percentile 2.5% | 0.02 | 0.01 | lower bound of the entire distribution negative values excluded |
| NoZeroDistribution Percentile 97.5% | 0.47 | 0.30 | upper bound of the entire distribution negative values excluded |
| ZeroDistributionStd | 0.13 | 0.08 | Std of the entire distribution negative values excluded |
| NoZeroDistributionMeanLow | 0.19 | 0.11 | lower bound of the mean negative values excluded |
| NoZeroDistributionMeanUp | 0.21 | 0.12 | upper bound of the mean negative values excluded |

**S1 Table**

**Reports the grand mean time course reliability with its accompanying confidence intervals - that were based on 488 observations - when negative values were included, negative values were omitted from the data. In addition, we give the 2.5% and 97.5% percentiles of the distribution and the standard deviation which were based on 1700 observations as well as the confidence interval of the mean which was likewise based on 1700 observations. All confidence intervals were obtained through (block) bootstrapping.**

**S2 Table. Reliability per brain region**

|  | mean reliability | std of reliability | mean realibaility AR(1) | std of relaibility AR(1) |
| --- | --- | --- | --- | --- |
| Left AIP | 0.20 | 0.13 | 0.12 | 0.08 |
| Left caudal IPS | 0.16 | 0.13 | 0.09 | 0.09 |
| Left dACC | 0.21 | 0.15 | 0.13 | 0.09 |
| Left FEF | 0.16 | 0.09 | 0.09 | 0.06 |
| Left IFG oper | 0.17 | 0.14 | 0.09 | 0.08 |
| Left IFG orb tria insula | 0.14 | 0.12 | 0.08 | 0.08 |
| Left IFS MFG IFG tria | 0.22 | 0.11 | 0.11 | 0.07 |
| Left IFS MFG | 0.29 | 0.20 | 0.15 | 0.12 |
| Left LIP | 0.12 | 0.12 | 0.06 | 0.06 |
| Left preCG1 | 0.12 | 0.10 | 0.06 | 0.06 |
| Left preCG2 | 0.18 | 0.13 | 0.11 | 0.08 |
| Left Precuneus | 0.23 | 0.17 | 0.13 | 0.10 |
| Left preSMA | 0.21 | 0.13 | 0.10 | 0.08 |
| Left PSPL | 0.16 | 0.11 | 0.10 | 0.08 |
| Left rostralMFG | 0.19 | 0.12 | 0.11 | 0.08 |
| Left SMG | 0.26 | 0.16 | 0.16 | 0.10 |
| Left STG MTG | 0.28 | 0.14 | 0.17 | 0.09 |
| Left VIP1 | 0.09 | 0.11 | 0.05 | 0.06 |
| Left VIP2 | 0.10 | 0.10 | 0.04 | 0.06 |
| Right AIP | 0.13 | 0.12 | 0.08 | 0.08 |
| Right caudal IPS | 0.16 | 0.13 | 0.08 | 0.07 |
| Right FEF | 0.15 | 0.10 | 0.08 | 0.06 |
| Right IFG orb insula | 0.16 | 0.10 | 0.08 | 0.06 |
| Right IFG tria | 0.19 | 0.15 | 0.11 | 0.09 |
| Right IFJ | 0.20 | 0.11 | 0.11 | 0.07 |
| Right LIP | 0.26 | 0.17 | 0.14 | 0.10 |
| Right midMFG | 0.31 | 0.14 | 0.17 | 0.09 |
| Right precuneus | 0.23 | 0.15 | 0.13 | 0.08 |
| Right preSMA1 | 0.24 | 0.14 | 0.12 | 0.09 |
| Right preSMA2 | 0.14 | 0.11 | 0.07 | 0.07 |
| Right PSPL | 0.12 | 0.10 | 0.08 | 0.07 |
| Right rostralMFG | 0.22 | 0.11 | 0.13 | 0.08 |
| Right VIP1 | 0.15 | 0.15 | 0.08 | 0.08 |
| Right VIP2 | 0.13 | 0.10 | 0.07 | 0.07 |

**S2 Table**

**Reports the mean within subject time course reliability per region without and with corrections for residual auto correlations AR(1) as well as standard deviation of the group.**

**S3 Table. Grand mean connectivity**

|  | Connectivity Test | Connectivity Retest | ConnectivityAR1 Test | ConnectivityAR1 Retest | DetectableConnectivity Test | DetectableConnectivity Retest | DetectableConnectivity AR1 Test | DetectableConnectivity AR1 Retest |
| --- | --- | --- | --- | --- | --- | --- | --- | --- |
| Raw mean connectivity | 0.41 | 0.40 | 0.33 | 0.32 | 0.16 | 0.16 | 0.09 | 0.09 |
| Percentage of negative paths | 0.31 | 0.26 | 0.21 | 0.22 | 0.34 | 0.29 | 0.22 | 0.22 |
| mean connectivity | 0.41 | 0.40 | 0.33 | 0.32 | 0.16 | 0.16 | 0.09 | 0.09 |
| lower bound connectivity | 0.31 | 0.29 | 0.24 | 0.24 | 0.08 | 0.08 | 0.03 | 0.03 |
| upper bound connectivity | 0.50 | 0.49 | 0.41 | 0.41 | 0.26 | 0.26 | 0.16 | 0.16 |
| Distribution percentile 2.5% | 0.04 | 0.03 | 0.03 | 0.03 | 0.00 | 0.00 | 0.00 | 0.00 |
| Distribution percentile 97.5% | 0.72 | 0.72 | 0.65 | 0.64 | 0.40 | 0.40 | 0.23 | 0.24 |
| lower bound of distribution mean | 0.40 | 0.40 | 0.33 | 0.32 | 0.16 | 0.16 | 0.09 | 0.09 |
| upper bound of distribution mean | 0.41 | 0.40 | 0.33 | 0.32 | 0.16 | 0.16 | 0.09 | 0.09 |
| STD of Distribution | 0.22 | 0.22 | 0.19 | 0.19 | 0.12 | 0.12 | 0.07 | 0.07 |
| lower bound of distribution STD | 0.22 | 0.21 | 0.19 | 0.18 | 0.11 | 0.12 | 0.07 | 0.07 |
| upper bound of distribution STD | 0.22 | 0.22 | 0.19 | 0.19 | 0.12 | 0.12 | 0.07 | 0.07 |

**S3 Table**

**Reports the grand mean time course connectivity with its** **accompanying confidence intervals - that were based on 488 observations – for** **conventional connectivity, conventional connectivity AR(1) removed, Detectable connectivity, Detectable connectivity AR(1) removed. In addition, we give the confidence intervals of the distribution which was based on 28050 observations as well as the confidence interval of the mean and std which were likewise based on 28050 observations. All confidence intervals were obtained through bootstrapping. The mean and its accompanying confidence intervals were made absolute when the CI upper bound was below zero. Mark that raw mean connectivity is the only statistic for which the mean was estimated from negative and positive correlations.**

**S4 Table. Random connectome statistics**

|  | **Connectivity** | **Connectivity AR(1)** | **Detectable** | **Detectable AR(1)** |
| --- | --- | --- | --- | --- |
| **mean conjunction** | 319 | 282 | 190 | 131 |
| **lower bound conjunction (2.5%)** | 274 | 244 | 128 | 79 |
| **upper bound conjunction (97.5%)** | 370 | 326 | 266 | 197 |
| **mean dice overlap** | 0.79 | 0.73 | 0.74 | 0.69 |
| **lower bound of dice overlap (2.5%)** | 0.74 | 0.69 | 0.66 | 0.61 |
| **upper bound of dice overlap (97.5%)** | 0.84 | 0.79 | 0.82 | 0.79 |
| **test mean connectivity** | 0.18 | 0.15 | 0.04 | 0.02 |
| **test lower bound connectivity (2.5%)** | 0.16 | 0.13 | 0.03 | 0.02 |
| **test upper bound connectivity (97.5%)** | 0.21 | 0.18 | 0.05 | 0.03 |
| **retest mean connectivity** | 0.15 | 0.12 | 0.03 | 0.02 |
| **retest lower bound connectivity (2.5%)** | 0.13 | 0.10 | 0.02 | 0.01 |
| **retest upper bound connectivity (97.5%)** | 0.18 | 0.15 | 0.05 | 0.03 |

**S4 table**

**Key statistics that were obtained from a bootstrapping procedure in which 10,000 random connectomes were created. We report the number of paths in conjunction, the height of the dice overlap measure, the height of the connectivity for, conventional connectivity, conventional connectivity corrected for residual auto correlations, detectable connectivity, detectable connectivity corrected for residual auto correlations as well as their confidence intervals.**

**S1 Figure. Brain behavior relation**


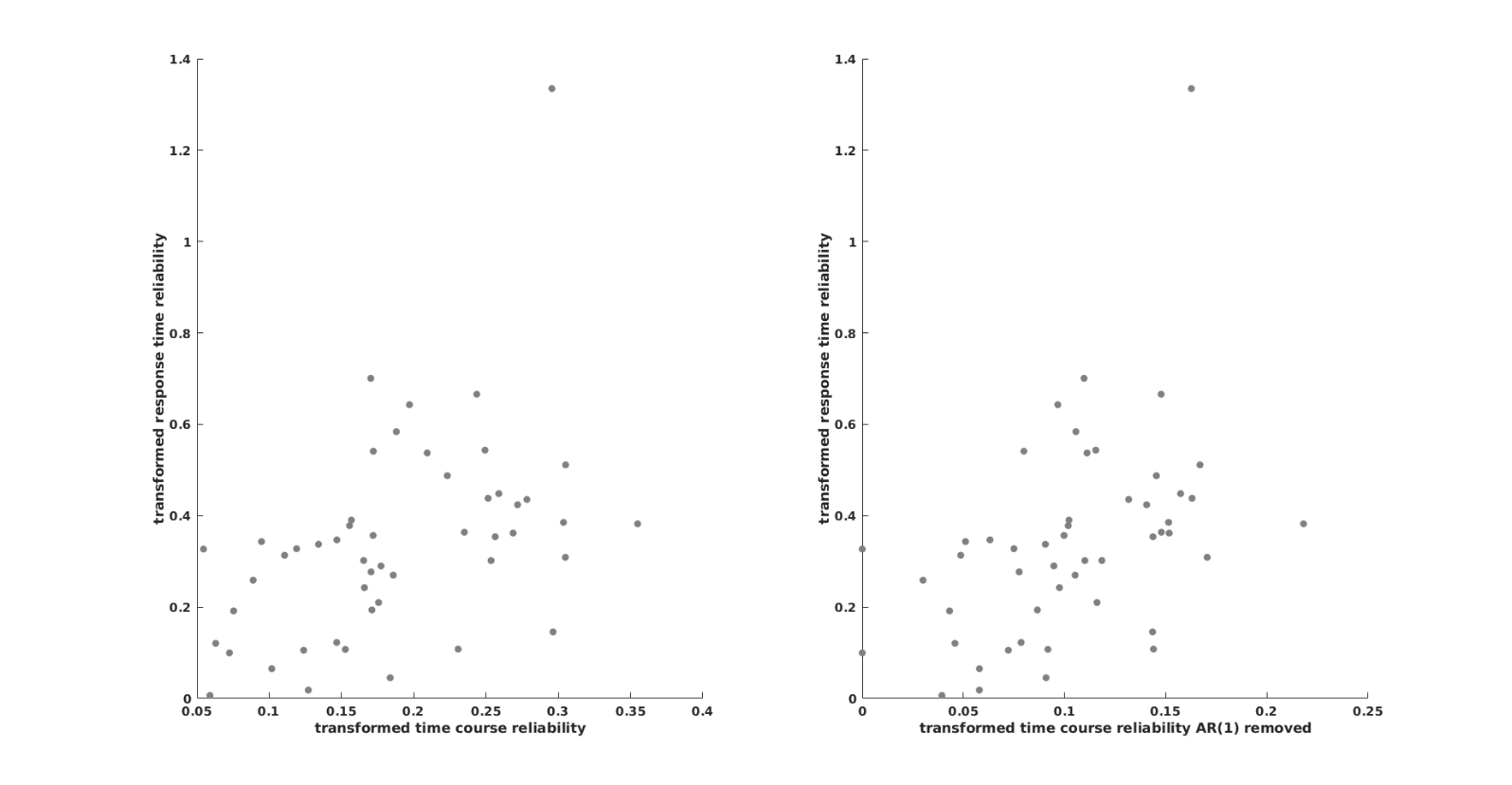


**S1 Fig.**

**In this graph we depict de relation between the mean time course reliability per subject and the response time reliability per subject.**

**S2 Figure. Histograms of t-statistics**


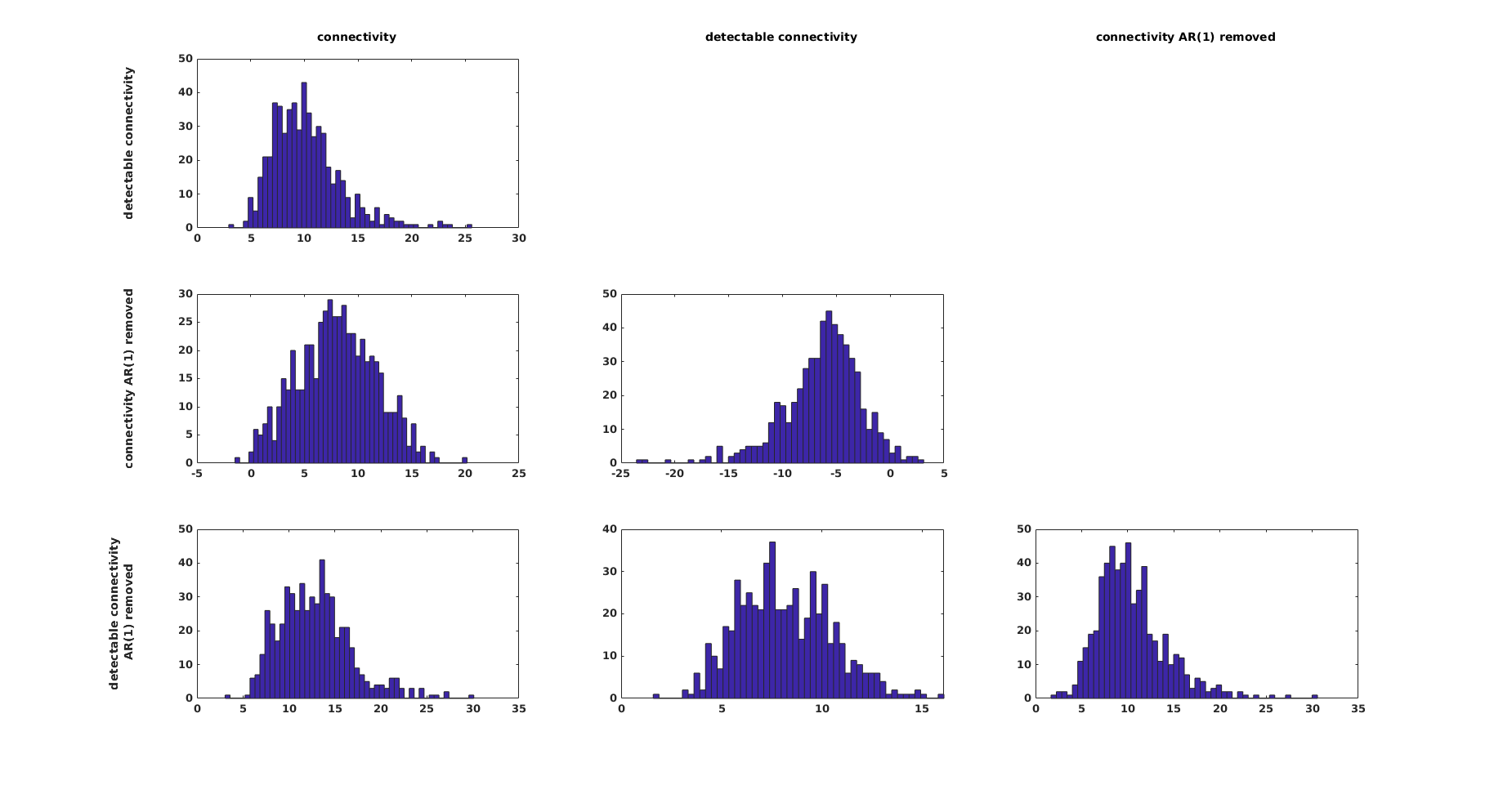


**S2 Fig.**

**We directly tested the four kinds of connectivity measures against each other resulting in six possible combinations. We took the smaller connectivity correlation of a test and retest run as a measure of conjunction. The 50 minimum correlations per path were tested against each other by means of a repeated measure t-test which resulted in 561 tests per combination. The vertical axis displays the number of paths that were detected for a specific combination. The horizontal axis reports the t-statistic. The results of the test procedures reveal large t-values. We estimated the median p value from the distributions for every single combination and give the results in the table below.**

|  | connectivity | detectable connectivity | connectivity AR(1) removed |
| --- | --- | --- | --- |
| detectable connectivity | 3.24E-13 |  |  |
| connectivity AR(1) removed | 2.33E-10 | 3.23E-07 |  |
| detectable connectivity AR(1) removed | 0* | 2.42E-10 | 3.27E-13 |

**^*)^ this value could not be estimated by matlab as it approached 0**

**S3 Figure. Scatter plots of connectivity**


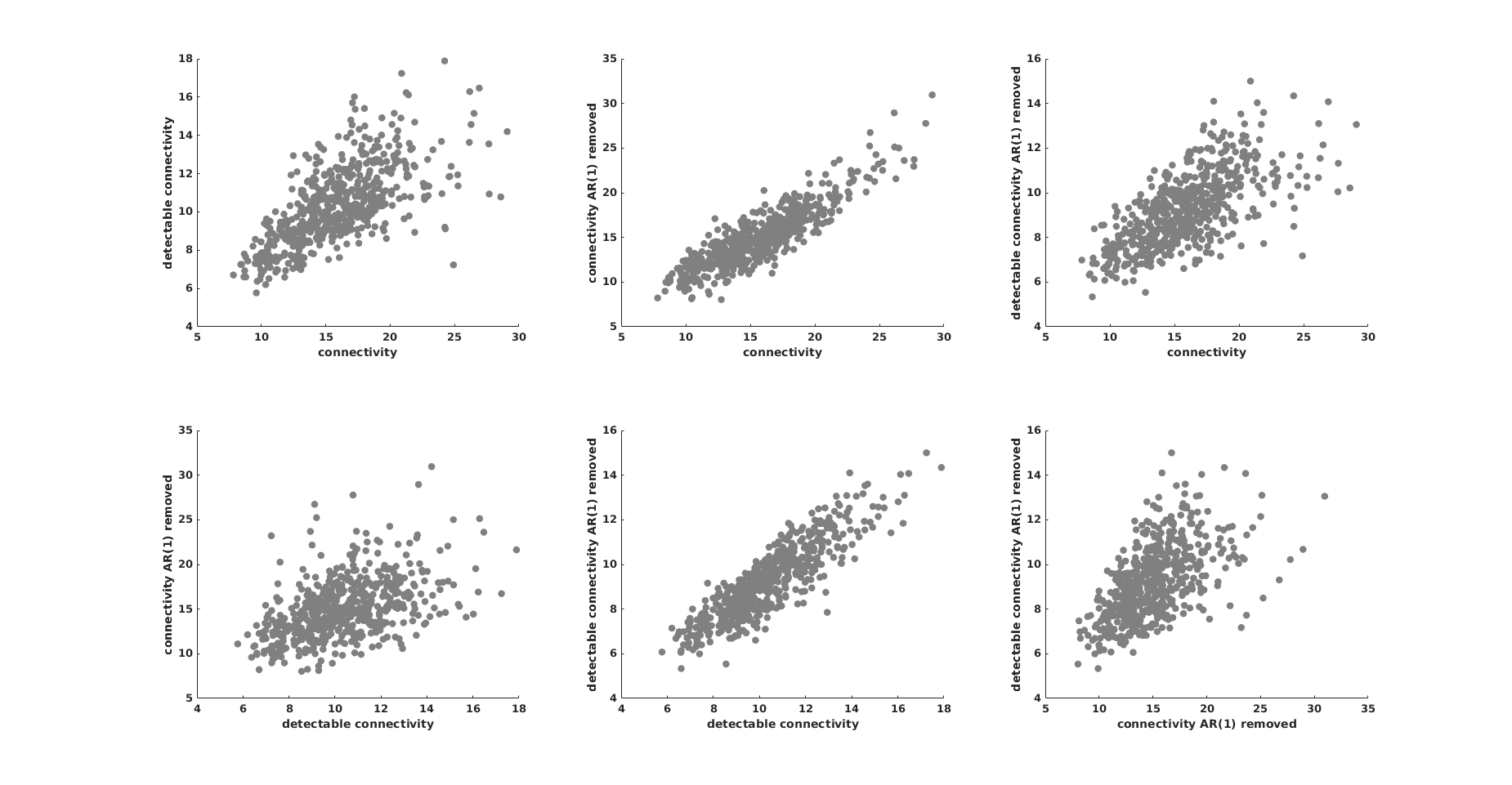


**S3 Fig.**

**561 paths of a test and retest run were subjected to NHST (n=50). Subsequently the smaller t value of the test and retest run was taken. The minimum t-values of the four kinds of connectivity measures were than directly scatterd against each other.**

**S4 Figure. Effect of corrections on connectivity maps**

|  |  |  |
| --- | --- | --- |
| **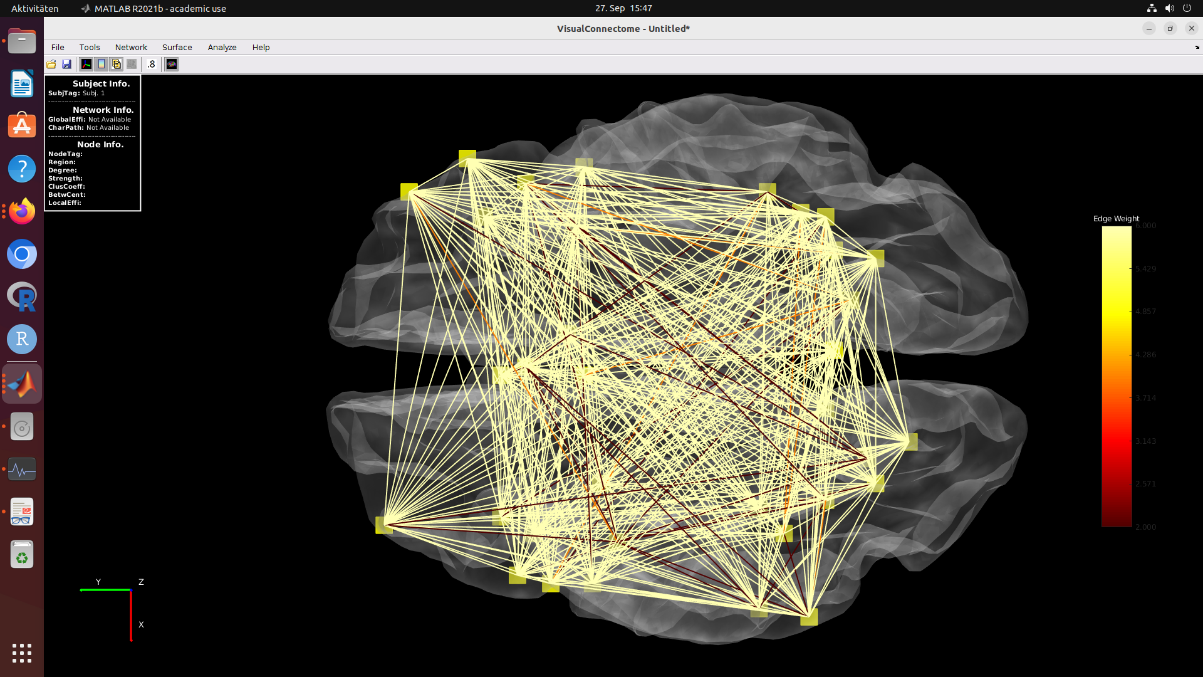** | **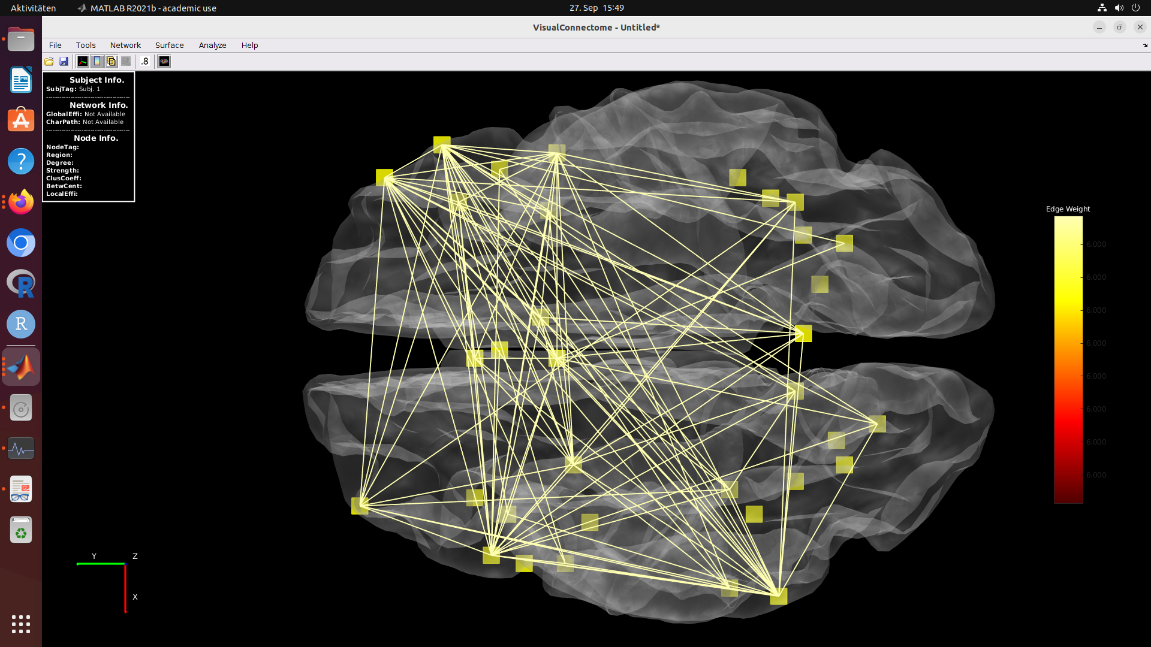** |  |
| **Connectivity** | **Detectable connectivity** |  |
| **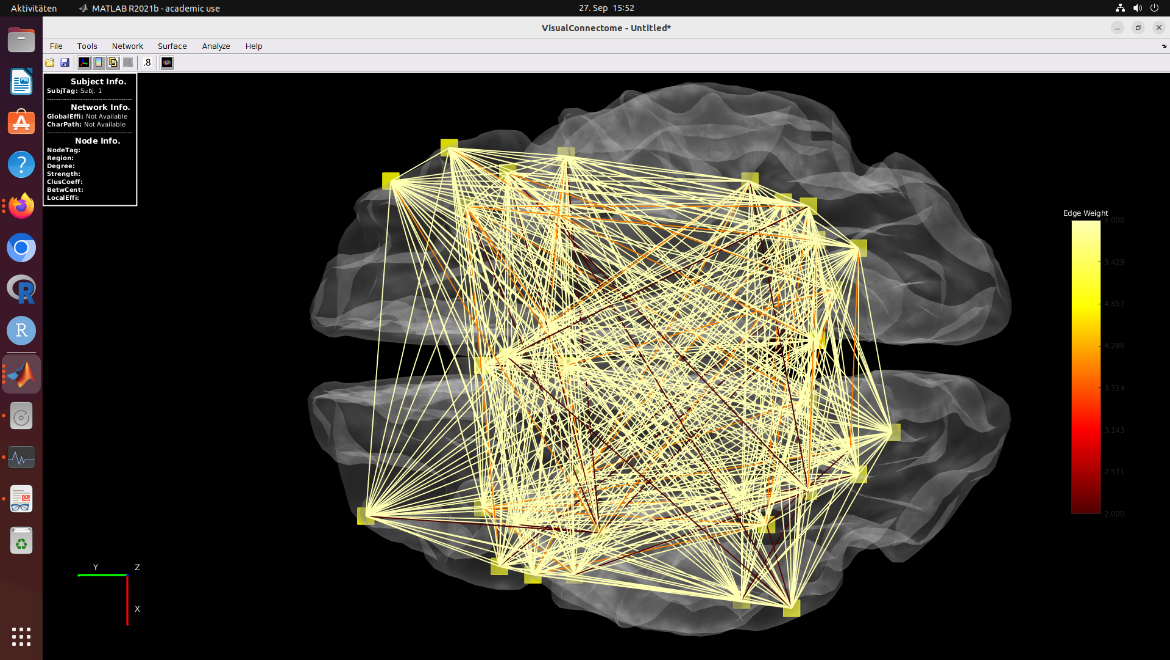** | **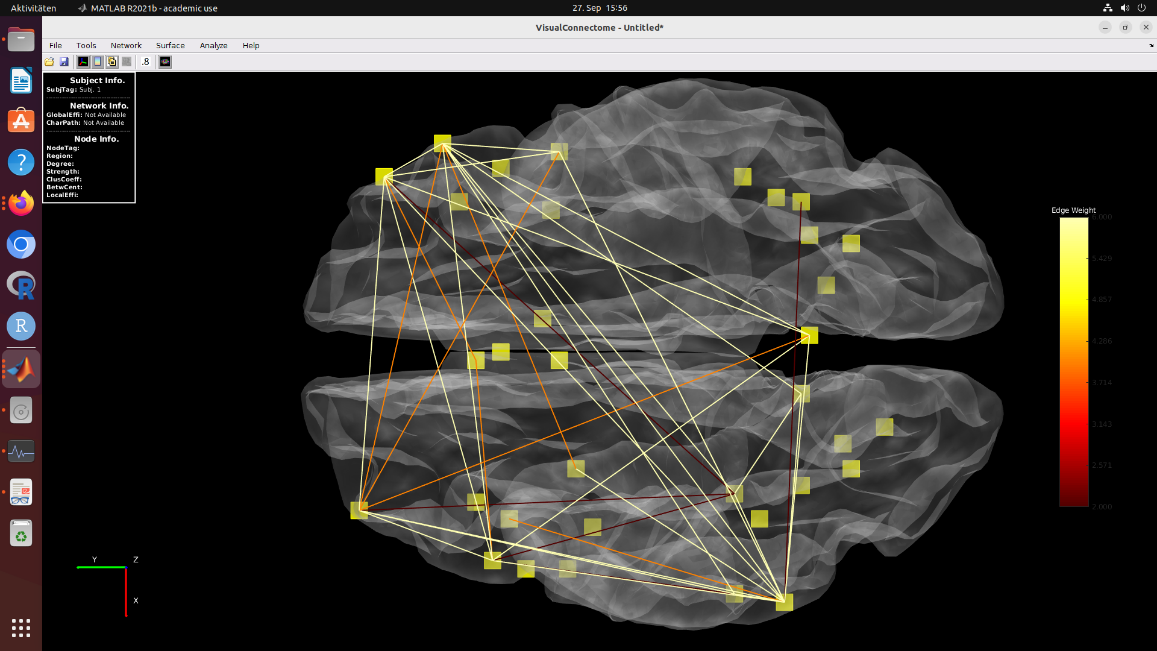** |  |
| **Connectivity corrected for AR (1)** | **Detectable connectivity corrected for AR (1)** |  |

**S4 Fig.**

**Here we visualize functional connectivity paths that were detected at p <1.69e^-16^. The 561 paths that were obtained from the four kinds of connectivity measures were subjected to NHST (n=50). Paths that were commonly detected in the test and retest run were depicted in yellow whereas paths that were exclusively found in the test or retest run were depicted in brown and orange respectively.**

**S5 Figure.** **Effect of corrections and sample size on the detected number of paths**


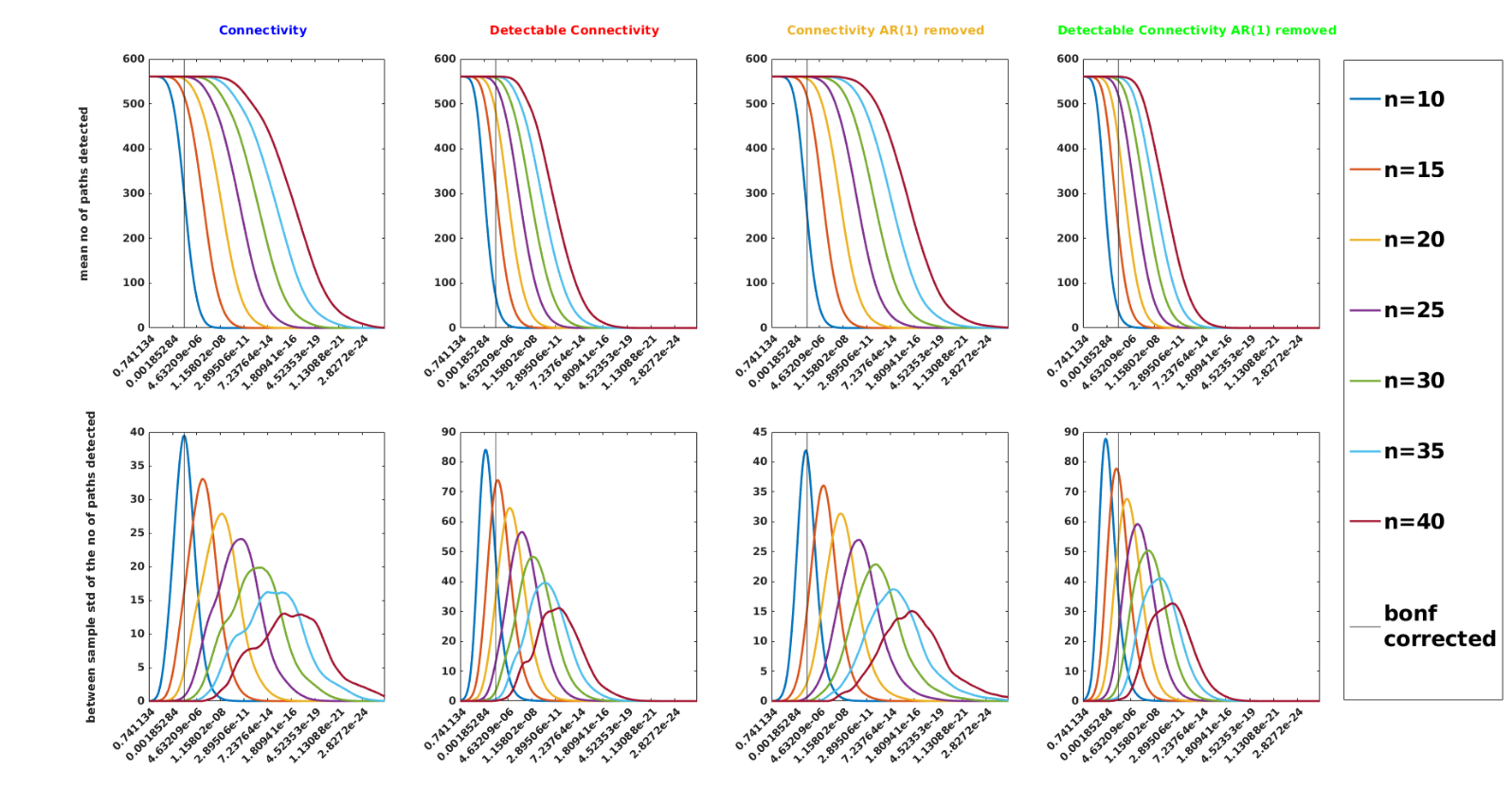


**S5 Fig.**

**The number of paths that survived conjunction analysis at a particular significance threshold were estimated for the four kinds of connectivity measures available within the context of a monte carlo simulation (10,000 iterations per sample size) which attempted to establish the relation between within sample reproducibility and sample size. Sample sizes varying between 10 and 40 are depicted with a specific color. The left most lines refer to sample sizes of 10 whereas the right most lines refer to sample sizes of 40.** **The vertical line refers to a Bonferroni corrected p value. For reasons of clarity, we only depicted sample sizes up to 40 as sample sizes above 40 do not deliver a lot of extra information.**

**The 561 paths of a test and retest run were subjected to an NHST based conjunction analysis at every single Monte Carlo iteration. The number of paths that were commonly detected at a particular significance threshold was estimated which resulted in 10,000 values from which standard deviations were estimated.**

**S6 Figure.** Effect of corrections and sample size on group reproducibility


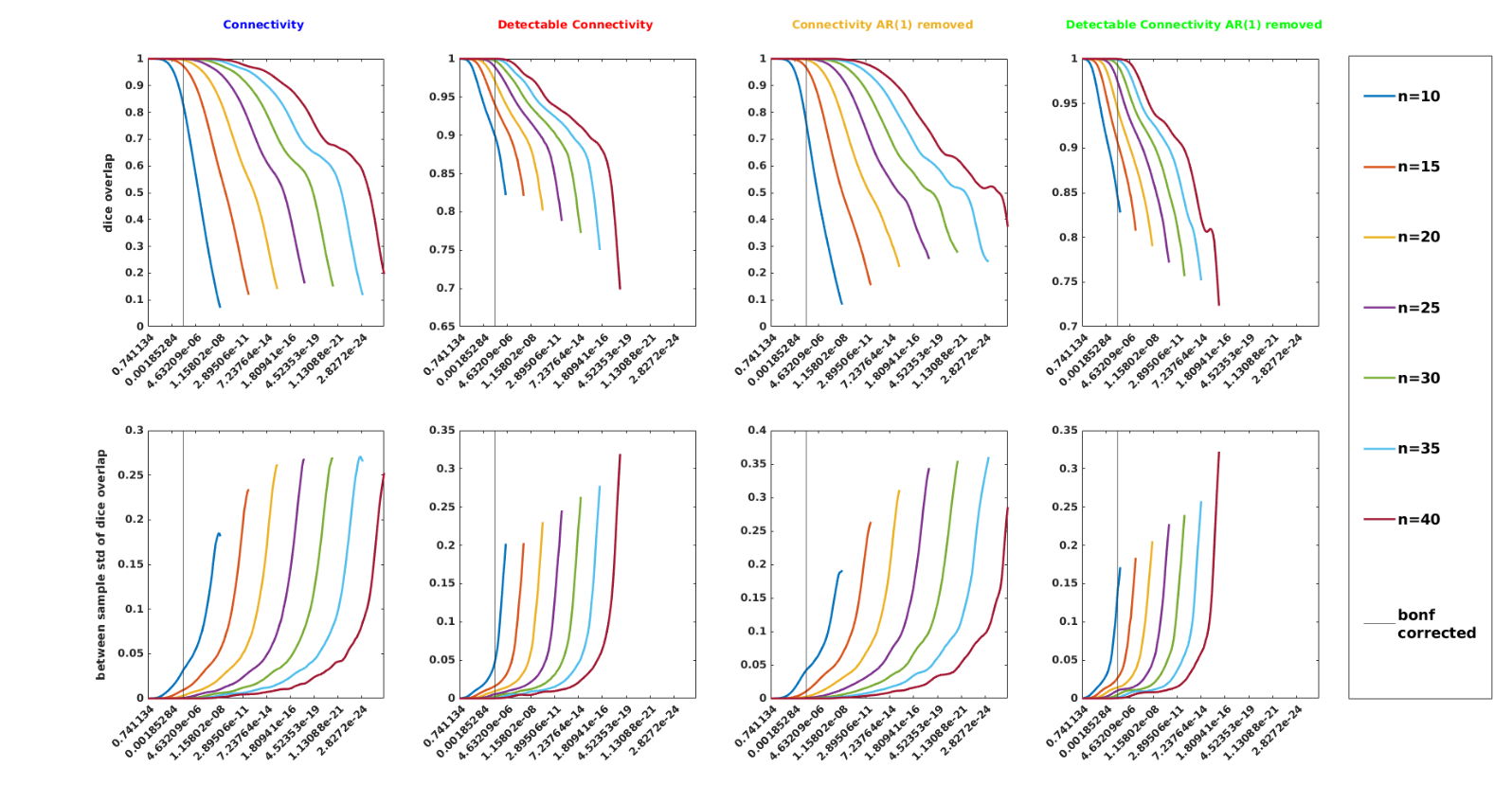


**S6 Fig.**

**The dice overlap that could be achieved at a particular significance threshold were estimated for the four kinds of connectivity measures available within the context of a monte carlo simulation (10,000 iterations per sample size) which attempted to establish the relation between within sample reproducibility and sample size. Sample sizes varying between 10 and 40 are depicted with a specific color. The left most lines refer to sample sizes of 10 whereas the right most lines refer to Distribution sizes of 40. The vertical line refers to a Bonferroni corrected p value. For reasons of clarity, we only depicted sample sizes up to 40 as sample sizes above 40 do not deliver a lot of extra information.**

**The 561 paths of a test and retest run were subjected to a dice overlap analysis at every single Monte Carlo iteration. The standard deviations were estimated from 10,000 dice overlap estimates that were obtained at a particular significance threshold.**

References

**1**. Koten JW, Langner R, Wood G, Willmes K. Are reaction times obtained during fMRI scanning reliable and valid measures of behavior. Exp Brain Res. 2013; 227:93–100. Epub 2013/04/07. doi: 10.1007/s00221-013-3488-2 PMID: 23564316.

**2**. Koten JW, Koschutnig K, Wood G. An attempt to model the causal structure behind white matter aging and cognitive decline. Sci Rep. 2023; 13:10883. Epub 2023/07/05. doi: 10.1038/s41598-023-37925-0 PMID: 37407647.

**3**. Fischl B, Sereno MI, Tootell RB, Dale AM. High-resolution intersubject averaging and a coordinate system for the cortical surface. Hum Brain Mapp. 1999; 8:272–84. doi: 10.1002/(sici)1097-0193(1999)8:4<272::aid-hbm10>3.0.co;2-4 PMID: 10619420.

**4**. Alakörkkö T, Saarimäki H, Glerean E, Saramäki J, Korhonen O. Effects of spatial smoothing on functional brain networks. Eur J Neurosci. 2017; 46:2471–80. doi: 10.1111/ejn.13717 PMID: 28922510.

**5**. Hagler DJ, Saygin AP, Sereno MI. Smoothing and cluster thresholding for cortical surface-based group analysis of fMRI data. Neuroimage. 2006; 33:1093–103. Epub 2006/10/02. doi: 10.1016/j.neuroimage.2006.07.036 PMID: 17011792.

**6**. Mikl M, Marecek R, Hlustík P, Pavlicová M, Drastich A, Chlebus P, et al. Effects of spatial smoothing on fMRI group inferences. Magn Reson Imaging. 2008; 26:490–503. Epub 2007/12/03. doi: 10.1016/j.mri.2007.08.006 PMID: 18060720.

**7**. Olszowy W, Aston J, Rua C, Williams GB. Accurate autocorrelation modeling substantially improves fMRI reliability. Nat Commun. 2019; 10:1220. Epub 2019/12/25. doi: 10.1038/s41467-019-09230-w PMID: 30899012.

**8**. Cronbach LJ, Hartmann W. A Note On Negative Reliabilities. Educational and Psychological Measurement. 1954; 14:342–6. doi: 10.1177/001316445401400213.
