# Supplementary material for: When most fMRI connectivity cannot be detected: Insights from time course reliability
Source: PLoS One. 2024 Dec 13;19(12):e0299753. doi: 10.1371/journal.pone.0299753 (PMC11642907; doi:10.1371/journal.pone.0299753)
Supplement: S1 Data — (ZIP) [file pone.0299753.s002.zip › PlosOneData/read me.docx]

This computer code was utilized to estimate the detectable connectivity based on the underlying reliability of the time courses in the 34 ROI by 50 subject time courses found in "fMRI_clean.mat". To execute the analysis, run "Estimate_ResAuto_TrueConBlockBoot.m". Ensure that the path where you saved the software is specified in the "current" variable. After running the code, the essential variables will be stored in memory. Not all variables are saved to conserve space, as it may be demanding for some computers. Once "Estimate_ResAuto_TrueConBlockBoot.m" is completed, run "bootRelSamplesize.m" to assess the impact of sample size on reproducibility.
