# Supplementary material for: When most fMRI connectivity cannot be detected: Insights from time course reliability
Source: PLoS One. 2024 Dec 13;19(12):e0299753. doi: 10.1371/journal.pone.0299753 (PMC11642907; doi:10.1371/journal.pone.0299753)
Supplement: Attachment — Submitted filename: Response to Reviewers_Koten_Ioannidis_PlosOne_Revision_S.docx [file pone.0299753.s003.docx]

*We would like to thank the reviewers for the many useful comments most of which we have tried to implement in the manuscript.*

**Presentation of Results: While the paper extensively describes the research methodology, it lacks any presentation of results. Ideally, the paper should include a results section detailing experimental findings and provide interpretation and discussion of these results.**

*The previous version of the manuscript consisted of sperate method and a result section. But they may have been difficult to distinguish, as the font size used for the titles was small. We have now made the fonts of the titles larger for clearer identification of where the method and result sections begin and end. The method section covers approximately 2600 words in the main text and around 2700 words in the supporting materials, while the results and its discussion section in the main text comprise around 3500 words.*

**Complex Statistical Methods: The paper employs some complex statistical methods, which may require further explanation and elucidation for better reader comprehension. Relating p-values to FDR correction to present statistical findings could enhance clarity.**

*We agree that the explanation of the methods is very technical and may need some extension as some of the methods employed are new in the field of neuroimaging. We have tried to introduce some of the novel methods in the introduction in a language that is more suitable to gain the attention of a larger public. We think that in a methods paper it is appropriate to introduce the basic principles of new methods in the introduction rather than in the methods section itself.*

*We discuss the imaging methods in use in higher detail in the supporting text S1 and S2.*

*We have extended a bit on the Flat-top lag-windows method in the main paper that is presented in the* “*Bootstrapping on the time course level” section and added:*

*“The flat-top lag-windows method involves choosing window sizes that are large enough to capture the autocorrelation structure of the time series, but small enough to avoid “averaging” over too many observations.”*

*You rightfully pointed out that FDR corrections are somehow standard in imaging. As for the FDR correction we have added some sentences in the “NHST based image reproducibility” section of the methods and added:*

*“All paths met the critical Bonferroni corrected p value even after conjunction analysis and stringent correction procedures, rendering more liberal methods like FDR corrections unnecessary. The Bonferroni thresholded data in our experiment automatically satisfies FDR correction criteria.”*

**Data Visualization: There is no mention of data visualization in the paper. Visualization tools can aid reader understanding of research findings. Therefore, incorporating charts or other visual elements to showcase key findings is recommended. Specifically, presenting statistical differences in brain network maps for a certain disease, such as ADHD (DOI: https://doi.org/10.1016/j.knosys.2022.109082) and PD (DOI: 10.1109/TCBB.2023.3252577), compared to a normal control group would be beneficial.**

***As for visualization:*** *We have now added a quotation in the paper of a software package that was used to visualize the connectomes that was developed by the* *Institute of Automation, Chinese Academy of Sciences. The package can be found at* [*https://code.google.com/archive/p/visualconnectome/*](https://code.google.com/archive/p/visualconnectome/)

***As for visual elements:*** *We have added an extra figure (now called figure 2) to the main paper that visualizes the four forms of connectivity including: connectivity, connectivity corrected for AR(1), Connectivity corrected for reliability, connectivity corrected for AR(1) and reliability. We hope that this figure summarizes all our findings comprehensively.*

*We are not quite sure if the reviewers saw all our figures that were uploaded together with the paper. Maybe something went wrong when downloading the paper from the internet. In total five figures were generated that in all cases consisted of at least three panels. In total roughly 30 visual elements were created for the main paper while a larger number of figures consisting of multiple panels are present in the supporting materials.*

***As for diseases****: We have mentioned the papers in the limitation section as they may open a window to novel ways of analysis. In which we state:*

*The Monte Carlo simulation clearly showed that a connectome that was based on hypothetical brain activation maxima derived from meta-analysis yields far more reproducible results compared to connectomes that have been selected randomly. However, this does not imply that meta-analysis derived connectomes are the best possible solution. As an alternative one could derive connectomes from hypothesis free methods such as independent component analysis, clustering and deep factor learning [31–33]. It was beyond the reach of this study to prove that connectomes derived from resting state and structural data by means of hypothesis free methods outperform brain regions that have been based on the neural correlates of working memory.*

*We acknowledge that our analysis is restricted to healthy individuals. And we report in the limitation section of the paper the following:*

*It is obvious that our data are limited to healthy individuals so that we cannot make any conclusions about clinical populations that may exhibit different within subject time course reliability.*

*However, there are no data from diseased populations that have been assessed using the same scanner parameters and working memory experiment as in our study.*

**Consistent Terminology Usage: Throughout the paper, consistency in the usage of concepts and terminology should be maintained to avoid reader confusion. For instance, if "within-subject time course reliability" is used in the text, it should be consistently utilized throughout the paper without substitution.**

*The reviewer correctly pointed out that we are somewhat inconsistent with the phrase "within-subject time course reliability". We have now corrected this issue. However, in a few cases we use the word time course reliability when the phrase within-subject time course reliability was used in the immediate context of the sentence to enhance readability.*

**Critical Reflection on Methods: When describing research methods, incorporating discussion on method limitations, such as potential biases in data collection or issues encountered in statistical analysis, can enhance the paper's comprehensiveness and reader understanding.**

*As for data collection: We have tried to raise a sample that is representative for the Austrian general population demographics. Moreover, we have applied rather strict selection criteria with regard to task performance and head motion so that the sample was substantially reduced.*

*As for limitations: we would like to point out that in the original paper several limitations were mentioned. We have now added some extra limitations and given the limitations section its own sub heading.*

*As for problems that were encountered in the data analysis: We in fact discussed in the supporting text S1 and S2 problems that were encountered. In these sections we reflect on the nature of negative reliability and its relation to auto correlation structure. We also reflect on the problems that we encountered when we had to deal with negative reliability in the context of averaging procedures. We have now added an additional analysis that reflects on the accuracy of AR(1) corrections.*

Reviewer #2: This study investigates the influence of within-subject time course reliability on the detection of connectivity and group effects using NHST, while accounting for variables such as sample size and spatial distribution of connectome nodes. The study performs a number of detailed analyses to tease apart the impact of these factors on the reliability and interpretability of functional connectivity measures in neuroimaging research. The paper is well written and makes an important point. To enhance clarity and the study's impact, I propose significant revisions to elaborate on these aspects. I have a few suggestions that could further enhance the clarity and impact of the study.

Major:

1. **The introduction section would benefit from a more explicit statement of the paper's objectives. While the aim to investigate the impact of within-subject time course reliability on connectivity detection and group effects is implied, a clearer articulation of the specific research questions or hypotheses would enhance reader understanding.**

*We have substantially extended the introduction in the hope that the research hypotheses are clearly formulated. As reported above we have now defined the four-research hypothesis in greater detail at the end of the introduction that reads as:*

*Here we test four hypotheses regarding the detectability of connectivity. We conducted a working memory experiment on two separate occasions and extracted two working memory-related functional brain networks. These networks were based on brain activity maxima identified in a previous large-scale meta-analysis that examined the neural correlates of working memory [20]. First, we investigated the effect of within-subject time course reliability on detectable connectivity. We hypothesized that connectivity among regions will be higher than the within-subject time course reliability. When observed connectivity exceeds the precision of time course measurement, we replace the connectivity correlation with the within-subject time course reliability correlation and refer to this as detectable connectivity. Second, we investigated the effect of within-subject time course reliability on null hypothesis significance testing (NHST) based group reproducibility. We expected that the mean detectable connectivity may be substantially lower than mainstream connectivity. Therefore, we hypothesized that mainstream group connectivity statistics obtained by testing connectivity correlations against zero are likely inflated. Third, we investigated the effects of sample size on group reproducibility. It is well established that power to detect group effects increases with larger sample sizes. We hypothesized that the differences between group statistics based on observed versus detectable connectivity will decrease with larger samples. Fourth, we investigated the effect of connectome selection on group reproducibility. It is reasonable to assume that a hypothetical connectome based on working memory-related brain coordinates may contain more robust brain activity data and therefore yield more reliable outcomes compared to randomly generated connectomes.*

2. **Additionally, the introduction occasionally suffers from a dense presentation of technical terms and concepts, which could potentially alienate readers unfamiliar with the intricacies of fMRI methodology.**

*We have tried to rewrite the introduction such that is more readable for a larger audience. We have tried to introduce the statistical concepts more carefully in the hope that it satisfies the reviewer and the audience. Key concepts that are needed to guide the reader trough the manuscript are printed in a cursive font after which they are defined.*

3. **Some of the methods suffer from slightly extreme language which is easily falsifiable, one can never ‘rule out the possibility” that “any lack” was due to poor performance or motion. Pease rework the text to be more balanced throughout.**

*We have reworked the text, as suggested.*

4. **The abstract mentioned 0.81 percent (is this correct, less than 1%?)…also where are the results that show this? It was not obvious from the paper.**

*In fact, the rationale was given in the beginning of the discussion where we stated that: “When corrected for residual auto correlations, the detectable amount of connectivity decreased even further to 0.09. Taking the square root of 0.09, we can infer that only 0.081 percent of the observed connectivity might be attributed to cognitive-related signal fluctuations”. We concur with the reviewer's assessment that the rationale was not adequately presented in the key section of the paper, specifically the abstract. Consequently, we have revised the abstract to enhance the clarity of the rationale behind this “slightly” shocking news.* *The key sentence in the abstract reads as follows: “The grand mean connectivity of the connectome equaled r =0.41 (95% CI 0.31-0.50) for the test run and r =0.40 (95% CI 0.29-0.49) for the retest run. The mean connectivity decreased to r = 0.09 (95% CI 0.03-0.16) when test-retest reliability and auto-correlations of single time courses were considered, indicating that less than a quarter of connectivity is detectable. The square root of the detectable connectivity r = 0.09 suggests that only 0.81% of the connectivity is explained by working memory-related time course fluctuations.”*

5. **Figures could be improved (e.g. figure on, needs legends for blue/reg plots for all panels)**

*We have added the legends.*

6. **Could the authors also plot the time course activity task effects? So not just the task-modulated connectivity, as these are measuring different things.**

*We are unsure of the reviewer's intended meaning. Figure 1D displays the grand mean fMRI time course, clearly indicating 24 signal changes that are associated with the 24 cognitive events being studied.*

**7. While the Discussion section effectively addresses the impact of within-subject time course reliability on connectivity measures, could the authors provide additional insights into potential strategies for mitigating the effects of variability in fMRI studies? For example, are there specific methodological approaches or analytical techniques that could help account for individual differences in time course reliability and improve the robustness of connectivity analyses?**

*As for individual differences we have now added the following passage:*

*We observed that very large individual differences in within subject response time reliability exist (range =0.01-0.87). These individual differences in response time reliability were correlated (r~0.45) with individual differences in within subject time course reliability. This finding corroborates previous observations that cognitive aspects of tasks are related to within-subject time course reliability [1].*

*As for improving robustness, we have just finished a paper that shows that within subject time course reliability can be doubled if low and high pass filters are carefully designed for a specific experiment. We have added a passage in the current paper in which we discuss several approaches that might work including filter optimization. We have added following passage to the paper:*

*What can be done to enhance within-subject time course reliability? A promising approach to improve within-subject time course reliability is to simply average task-related time courses obtained from multiple independent fMRI sessions. Alternatively, one might incorporate several time courses of the same individual that measure the same task into a multivariate statistical model such as structural equation modeling. Such multivariate techniques have been shown to improve reliability on the group level [5]. A cheaper alternative might be to refine high and low-pass filters used to separate noise from signal. Current fMRI software packages typically employ a one-size-fits-all filter approach, which may not be optimal. Theoretically, designing filters based on the expected shape of the task-related response could effectively separate noise from signal without excessively altering the autocorrelational structure of the time courses. Possibly ultra-high field scanners with more favorable signal to noise ratios could detect higher levels of within-subject time course reliability. Within this context, it would be beneficial to assess the contributions of signal-noise ratio under different measurement conditions and post-processing techniques through a sensitivity analysis.*

**8. Additionally, considering the limitations discussed, do the authors have any recommendations for future research aimed at addressing these challenges and further validating these findings across diverse experiments. It may be beneficial to explore the potential implications of considering whole-brain functional connectivity in addition to the specific regions investigated in this study.**

*As for whole brain analysis: So, focusing on a small connectome is indeed a short coming of this study that we discuss in the limitation section of this paper. It is in principle feasible to estimate the detectable connectivity in a whole brain fashion by taking one time course form the test sessions as a seed and all the time courses of the retest session as the target time courses. One can than loop through all the seed time courses of the test session and the respective time courses of the retest session and average the result. While this is certainly a time-consuming operation is not impossible on modern computers. We have added the following sentence to the limitations section.*

*“It is obvious that there is a need for studies that estimate the detectable connectivity of task-based fMRI on the whole brain level. While this potentially computationally expensive it is not impossible.”*

**9. The drastic reduction in average connectivity and decrease in detectable connectivity after correcting for residual autocorrelations needs further validation and replication of the findings to ensure the robustness and reliability of the conclusions drawn from the data. What are potential confounding factors in connectivity analyses and how corrections reflect true connectivity levels requires careful consideration.**

*We have added the following sentence in the discussion: “The drastic reduction of within-subject time course reliability after AR(1) corrections needs to be replicated in future studies as it has not been observed so far.“*

*AR(1) corrections are standard in all software packages currently employed in neuro imaging. We share our doubts with the reviewer whether one should indeed only rely on these corrections which is the reason why we also performed block bootstrapping that gives correct confidence intervals despite the very high auto correlations in the data. However, block bootstrapping while common in the economical sciences is to best of our knowledge only very rarely applied in the brain sciences maybe because they are computationally expensive and difficult to perform on the whole brain level. According to our expert Hans Manner, in the economical sciences the debate between auto correlation corrections versus block bootstrapping has not yet been solved and maybe it is about time to also open the debate in the brain sciences. Running 10000 simulations per path per subject is however no mean feat. I took us roughly 2 days to perform block bootstrapping on a parallel computing system with 10 processors in our rather small connectome consisting 561 paths*50 subjects only.*

**10. Furthermore, the authors are using anatomically based/fixed ROIs and thus voxels within the ROIs may not be temporally coherent. They thus might consider at least discussing the potential utility of data-driven/probabilistic parcellation/decomposition approaches, such as independent component analysis (ICA), for estimating brain components and their connectivity, or using one of the existing FC+clustering parcellation approaches. The rationale behind connectome selection and its potential impact on connectivity measures and group-level analysis should be highlighted. Glasser, Matthew F., et al. "A multi-modal parcellation of human cerebral cortex." Nature 536.7615 (2016): 171-178. Du, Yuhui, et al. "NeuroMark: An automated and adaptive ICA based pipeline to identify reproducible fMRI markers of brain disorders." NeuroImage: Clinical 28 (2020): 102375.**

*We have added the following text to the limitation section and added the suggested literature.*

*The Monte Carlo simulation clearly showed that a connectome that was based on hypothetical brain activation maxima derived from meta-analysis yields far more reproducible results compared to connectomes that have been selected randomly. However, this does not imply that meta-analysis derived connectomes are the best possible solution. As an alternative one could derive connectomes from hypothesis free methods such as independent component analysis, clustering and deep factor learning [31–33]. It was beyond the reach of this study to prove that connectomes derived from resting state and structural data by means of hypothesis free methods outperform brain regions that have been based on the neural correlates of working memory. However, we think that our approach to use brain activation maxima that have been obtained through working memory experiments is very appropriate if one analyzes a working memory experiment. Classic task-based fMRI studies operate on the assumption that the Hemodynamic Response Function is correlated with the time courses being analyzed. When time course alpha and beta both correlate significantly with a predictor time course p, which is based on the HRF, this implies that alpha is correlated with beta. Therefore, task positive brain activation maxima as identified in classic task-based fMRI studies are inherently functionally linked to one another. So, we respectfully disagree with the reviewer and have added following text in the discussion.*

*Our Monte Carlo analysis revealed that detectable connectivity and group reproducibility is affected by the exact coordinates of the nodes that are used to construct the connectome. The simulations demonstrate that the hypothetical connectomes derived from a working memory meta-analysis is significantly more reliable when compared to the 10,000 randomly generated connectomes. As an example, we want to mention that the average connectivity of the hypothetical connectome was 0.4 while it was only 0.18 (test) and 0.15 (retest) for random connectomes. The detectable connectivity for the hypothetical connectome was r = 0.09 when time courses were corrected for within subject time course reliability and auto correlations while it was r=0.02 when connectomes were randomly selected. The latter difference does not seem to be impressive but it has nonetheless far-reaching consequences for the group reproducibility of the images on the basis of conjunction analysis. While all 561 paths of the hypothetical connectome were reproduced at a Bonferroni corrected p value after rigorous corrections, only 131 paths were reproduced when connectomes were randomly selected. This demonstrates that there is some merit in the use of meta-analysis results when constructing hypothetical connectomes. The effectives of the hypothetical connectome were in particular visible in supporting Figure S4 were a p <1.69^e-16^ was needed to differentiate group statistic maps from each other. We conclude that hypothetical connectomes that are derived from brain activation studies are also relevant for connectivity studies.*

*Ultimately, we wish to emphasize that the primary issue in this study lies not in the connectivity between regions, as this connectivity can indeed be substantial as can be viewed in figure 2. Instead, the key concern is the persistently low level of reliability, even in areas with high connectivity. Therefore, enhancing connectivity does not necessarily lead to improved reliability.*

**11. Similarly, the incorporation of 34 MNI coordinates for reading from external data is suboptimal to using functional localization in held out data, this will reduce task effects and should be noted as a limitation. Techniques such as selecting upper quartile or other voxel subsets within ROIs can help, but are also a bit tricky to implement without inducing bias.**

*We agree with reviewer that reading external data will reduce task effects in the held-out data which is exactly the reason why we did this. The main goal is that you want to avoid circularity as this may result in the so-called voodoo phenomena that occur when maxima, quartiles etc. etc. are extracted from the same data as the data that are analyzed.*

**12. Additionally, the choice of an 8 mm radius needs further discussion. Was this a ‘smooth	with’ or ‘smooth to’ 8 mm? How sensitive are the results to the smoothing kernel?**

*”We agree with the reviewer that the rationale behind our choice was not fully described in the supporting methods and we have now updated it and added extra literature. So, we have extensively discussed this matter in the supporting text S1 under the heading “Alignment” and “Creation of Patches of Interest and Extraction of Grey Matter Time Courses*

**13. Averaging reliability estimates across paths may oversimplify the assessment of reliability, particularly if there is substantial variability across different paths. Individual paths may exhibit different levels of reliability and averaging them together could obscure important patterns in the data and averaging across participants may overlook this variability.**

*We completely agree with the reviewer which is the reason that in the previous manuscript we showed figure 1F. Figure 1F displays the entire range of reliabilities as observed for the individual subjects under study per region. You can for instance see that the right MFG has the highest average test-retest reliability while it nonetheless shows a very considerable variability in the individual reliabilities. In addition, we also reported the average test-retest reliabilities and standard deviations as obtained from the various methods per brain region in the supporting material table 2. It is possible that the axes in figure 1F are hard to read in the uploaded document as it is not depicted in high resolution. However, believe us it is there and the low resolution in the upload is not of our wrong doing as we uploaded high resolution version of it. We will do our best to convince Plos One to plot it in high resolution such that you are able to see it.*

**14. The appropriateness of the AR(1) model depends on the underlying structure of the data, and studies have argued that AR(1) is not a sufficient model for neuroimaging data. In addition, given the autocorrelation is strongly related to the hemodynamic response function, which can vary in interesting ways across populations, it may also be important to evaluate the autocorrelation for such effects. These issues could be discussed in the manuscript.**

*Although AR(1) models are standard, maybe they are not the best solution. As mentioned in the paper we estimated the weights that were used for the correction procedure per subject per path/node which should account for some of the individual differences that may occur. However, is the AR(1) model truly appropriate? In our study the answer is most likely yes. We have added a new analysis in the supporting methods that tries to demonstrate this. In a first step we analyzed the auto correlation functions of the denoised data that despite denoising show very high autocorrelation as is unfortunately usually the case in fMRI. Next, we analyzed the auto correlation function of the data that were corrected with an AR(1) model. Very tiny autocorrelations remained at lag 1 to lag 3 that in no instances exceed r = 0.05. So, the detectable connectivity is possibly still slightly overestimated although we think the effect is not traceable. Imposing more complicated ARMA models did not seem to really help so our expert in the field Hans Manner suggested to stick with the usual AR(1) model as it did a rather decent jab as can be seen from the additional analysis provided in the supporting text 1 where the more complicated aspects of the imaging procedures are given.*

**15. How do these findings generalize to other datasets? Robustness checks and sensitivity analyses can help assess the stability of the results across different conditions. The reproducibility of the results should be discussed in manuscript. Do other brain regions or functional networks analysis support these findings?**

As for generalization we have added the following sentences.

*How do our results generalize to other studies and populations? Our study demonstrated complete group connectome reproducibility at P < 2.54e-6 even when stringent corrections were applied. The nodes utilized in this investigation were originally derived from a working memory study, suggesting that findings from brain activation studies can be generalized to connectivity studies across distinct samples. The highest mean within-subject time course reliability of a specific region was slightly higher in this study (r=0.31) compared to the study of our colleagues (r=0.25) [1]. Whether this is a fair comparison is debatable since field strength and task designs were different. The findings from our group reliability analysis suggest good (0.64 to 0.73) reliability which surpass expectations based on recent fMRI meta-analyses which estimate that group reproducibility of fMRI does usually not exceed r= 0.4 [3,4]. As the resting state time courses obtained from a test and rest run are inherently not synchronized due to the wandering nature of the mind, it is impossible to make any meaningful statements about the detectable connectivity of resting state studies. This draws a cast on the clinical applicability of this attractive technique in the context of pre surgical mapping and other critical applications of fMRI. It is obvious that our data are limited to healthy individuals so that we cannot draw any conclusions about clinical populations that may exhibit different within subject time course reliability. In our view, it is important to examine the within-subject time course reliability of language and hand functions using robust block designs, as these functions are essential for effective pre-surgical mapping.*

Minor:

1. The manuscript would benefit from a clearer separation between the methods and results sections. Specifically, it appears that certain aspects of the results, such as the content found in the last paragraph of page 13, contain information that would be more appropriately situated within the methods section.

*We have provided a more clean separation of Methods and Results, as above. We have added some lines on thresholding policies in the methods section*

________________________________________
